# Type I Interferons and Cancer: An Evolving Story Demanding Novel Clinical Applications

**DOI:** 10.3390/cancers11121943

**Published:** 2019-12-04

**Authors:** Eleonora Aricò, Luciano Castiello, Imerio Capone, Lucia Gabriele, Filippo Belardelli

**Affiliations:** 1 FaBioCell, Core Facilities, Istituto Superiore di Sanità, 00161 Rome, Italy; eleonora.arico@iss.it (E.A.); luciano.castiello@iss.it (L.C.); 2 Department of Oncology and Molecular Medicine, Istituto Superiore di Sanità, 00161 Rome, Italy; imerio.capone@iss.it (I.C.); lucia.gabriele@iss.it (L.G.); 3 Institute of Translational Pharmacology, Consiglio Nazionale delle Ricerche, 00133 Rome, Italy

**Keywords:** type I interferon, cancer, immunotherapy, dendritic cells

## Abstract

The first report on the antitumor effects of interferon α/β (IFN-I) in mice was published 50 years ago. IFN-α were the first immunotherapeutic drugs approved by the FDA for clinical use in cancer. However, their clinical use occurred at a time when most of their mechanisms of action were still unknown. These cytokines were being used as either conventional cytostatic drugs or non-specific biological response modifiers. Specific biological activities subsequently ascribed to IFN-I were poorly considered for their clinical use. Notably, a lot of the data in humans and mice underlines the importance of endogenous IFN-I, produced by both immune and tumor cells, in the control of tumor growth and in the response to antitumor therapies. While many oncologists consider IFN-I as “dead drugs”, recent studies reveal new mechanisms of action with potential implications in cancer control and immunotherapy response or resistance, suggesting novel rationales for their usage in target and personalized anti-cancer treatments. In this Perspectives Article, we focus on the following aspects: (1) the added value of IFN-I for enhancing the antitumor impact of standard anticancer treatments (chemotherapy and radiotherapy) and new therapeutic approaches, such as check point inhibitors and epigenetic drugs; (2) the role of IFN-I in the control of cancer stem cells growth and its possible implications for the development of novel antitumor therapies; and (3) the role of IFN-I in the development of cancer vaccines and the intriguing therapeutic possibilities offered by in situ delivery of *ex vivo* IFN-stimulated dendritic cells.

## 1. Introduction

Since the publication of the ground-breaking study by Ion Gresser in 1969 showing the antitumor effect of IFN-α/β (IFN-I) in mice [1], the role of these cytokines in cancer has been the topic of investigation for the subsequent 50 years. Further studies in mouse models by many groups, including ours, opened the path to clinical experimentation, firstly using natural IFN-α and later recombinant IFN-α 2 subtypes [2]. For many years, the antitumor effects observed in patients with hematological malignancies (hairy cell leukemia and chronic myeloid leukemia) and solid tumors, including melanoma and renal cancer, maintained the elevated attention of patients and the media on IFN-I.

Today, many oncologists consider IFN-I as “dead drugs”, replaced by new tools and protocols. It should be noted, however, that IFN-I were used in cancer patients when their mechanisms of action were largely unknown, as either conventional cytostatic drugs or non-specific biological response modifiers. They were generally utilized at high dosages and administered continuously. Biological activities subsequently ascribed to these cytokines were barely considered for clinical use. Notably, relevant toxicity is mainly associated with high dosage IFN treatments in cancer patients [2,3]. Serious adverse effects of systemic IFN regimens include flu-like symptoms, fatigue, anorexia, elevated liver enzyme levels, myelosuppression, and neurologic symptoms. These symptoms can be managed by appropriate pharmacological intervention, but such a relevant toxicity can ultimately lead to premature discontinuation of IFN treatments [2,3]. Retrospectively, we can say that hasty and massive clinical use of IFN-I in cancer, coupled with excessive expectations of efficacy for a single drug, have contributed to the perception of failure for IFN-I as clinical drugs. Industries involved in the production of recombinant IFN-I now have poor interest in exploring novel modalities of clinical use, as they prefer investing in new drugs. Nevertheless, the history of IFN-I as antitumor drugs remains an example of therapeutic survival in the face of several cycles of optimism and discouragement. In fact, fifty years after the first demonstration of an antitumor effect of IFN-I in mice [1], therapeutic opportunities from new insights stemming from the progress in cytokine and cancer research have been underlined [2,4].

As of November 2019, Clinicaltrials.gov lists more than 140 (recruiting or active) clinical trials based on the use of IFN as well as of agents/formulations designed for either inducing endogenous IFN signals or for the in situ delivery of the cytokine. Most of these studies involve IFN-based strategies in combination with other conventional or novel protocols, thus testifying the vital interest still existing in the IFN-I system for the development of clinically more effective combination therapies.

Much of the data in humans and mice underlines the importance of endogenous IFN-I, produced by both immune and tumor cells, in the control of tumor growth [5,6,7] and in the response to antitumor therapies [8,9,10,11]. In many cases, the presence of an IFN-I signature in the tumor microenvironment (TME) is a marker of the so-called “hot tumors” (i.e., tumors exhibiting specific immune cell infiltrates predicting a potential therapeutic response). In contrast, the suppression of an IFN-I system, at the transcriptional level or by effect of receptor downregulation, is often associated with a worse clinical outcome and increased tumor progression [6,7,12].

While the research progress underscores new opportunities to exploit the knowledge on the IFN-I system for the development of biomarkers of response to antitumor therapies, recent studies reveal new targets and mechanisms of action. There are recent and excellent review articles on the antitumor effects of IFN, where emerging areas, such as that of the clinical use of activators of the stimulator of IFN genes (STING) pathway and other endogenous IFN signals have been reviewed and discussed [2,4,13]. However, some of the new mechanisms of action and therapeutic opportunities regarding the use of IFN have not yet been discussed. In the present Perspective Article, we will restrict the focus on these, so far poorly considered, new or unexplored mechanisms, which may be relevant for designing novel modalities for a local use of IFN-I potentially capable of resulting in more effective and selective anticancer therapies.

**Figure 1:**
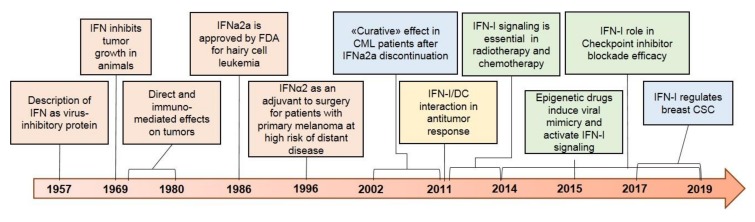
The major milestones of IFN-I research, here shown in orange, resulted in IFN-α being the first immunotherapeutic drug approved by the FDA, at a time when their mechanisms of action were not fully unraveled. Despite initial enthusiasm, clinical use of IFN-I in cancer has now been largely replaced by novel targeted therapies. The accumulated evidence of the last ten years, enlisted in colors on the right side, suggests a rethinking of the prominent role of IFN-I in determining cancer development, progression, and response to therapy. The importance of IFN-I signaling emerged as crucial for response to chemotherapy, radiotherapy, immune checkpoint inhibitor therapy, and epigenetic drugs (shown in green). Moreover, in vitro and clinical observations (shown in blue) from chronic myeloid leukemia (CML) and, more recently, breast cancer highlighted IFN-I as regulators of cancer stem cell proliferation and differentiation. Lastly, IFN-I emerged as essential for the ability of dendritic cells (DC) to activate antitumor immunity (in yellow).

Figure 1 illustrates key historical events in the clinical development of IFN-I and some recent and important discoveries leading to novel perspectives in the clinical use of these cytokines in cancer patients.

## 2. IFN-I in Combination Therapies

### 2.1. IFN-I and Chemo-Radiotherapy

A growing body of literature indicates that the antitumor response induced by chemotherapy and radiotherapy relies, at least in part, on the activation of IFN-I in cancer, as well as in immune cells (reviewed in [14]). In particular, data in animal models suggest that IFN-I can modulate the immunogenicity of cell death induced by certain cytotoxic anticancer treatments. As an example, cisplatin, a drug unable to induce immunogenic cell death (ICD), fails to induce a protective antitumor immunity in tumor bearing mice, unless it is preceded by the intratumoral injection of IFN-I [8]. IFN-I can also represent an added value in combination with chemotherapy agents known to induce IFN-I and ICD: mice bearing transplantable lymphomas treated with cyclophosphamide and IFN-I showed a synergistic antitumor response by mechanisms including ICD induction and activation of dendritic cells (DC), and subsequent cross-presentation of tumor antigens to CD8+ T cells [15]. These data, as well as others (reviewed in [14]), lead to the concept that IFN-I can synergize not only with chemotherapy, but also with radiotherapy by multiple mechanisms, acting on apoptosis, ICD and immune cells. Since the results of preliminary studies combining IFN and chemo/radiotherapies were encouraging, but hampered by toxicity [16], in reconsidering how IFN-I should be used in new generation combination therapies, a particular focus is needed on timing and dose administration. A discontinuous use of IFN-I could ensure a transient and acute exposure of TME to the cytokine, that will most likely promote ICD and DC activation, while avoiding not only toxicity, but also phenomena, such as down-regulation of IFN-I receptors and possible immune suppression induced under a chronic activation of the IFN-I system [17].

### 2.2. IFN-I and ICI

Immune checkpoint inhibitor (ICI) therapy represents a pillar of current therapies for advanced cancer [18]. However, despite their impressive efficacy, treatment failure is frequently observed and resistance and relapse are common. This phenomenon relies on both tumor-cell intrinsic and extrinsic factors in the TME [19]. Of note, loss-of-function mutations or alteration in the IFN-I signaling pathway have been associated both with immune escape [6,12,20] and impaired response and/or resistance to ICI [11,21,22,23]. Interestingly, in the TME of both mice and patients with triple negative breast cancer (TBNC), IFN-I signaling has been shown to decrease with age, and this associates with impaired response to ICI therapy that can be restored with a STING agonist, an IFN-I inducer [24]. However, while a positive correlation between IFN-I signaling activation and ICI has been demonstrated, prolonged IFN-I signaling may favor a resistance program to ICI [10]. Notably, the combination of IFN-α, in its pegylated form, and ICI has been tested in melanoma and renal cell carcinoma [25,26], but results were hampered by the toxicity of the continuous administration of peg-IFN-α. We propose that conveniently targeting tumor-inherent IFN-I signaling by discontinuous treatment can offer a new therapeutic opportunity to overcome primary or acquired resistance to ICI.

### 2.3. IFN-I and Epigenetics

In cancer cells, IFN-I may act within a state of “viral mimicry” which is characterized by the accumulation of cytosolic DNA induction, activation of the cyclic GMP-AMP synthase (cGAS)/stimulator of IFN genes (STING) pathway, reactivation of immunogenic cancer antigens, increased HLA-class I-restricted antigen presentation, and downstream production of IFN-I and pro-inflammatory cytokines [27,28]. Of interest, “viral mimicry” has been shown to be induced by epigenetic inhibitors (EPIi), including DNA methyltransferase inhibitors (DNMTi) and histone deacetylase inhibitors (HDACi) [29]. EPIi highly modulate the tumor microenvironment, reducing immunosuppressive signals through activation of IFN-I signaling [30]. These evidences highlight the crucial role of epigenetic modulation in the constitutive and induced expression of IFN-I and IFN-stimulated genes [31]. Given their potent antitumor efficacy in some hematological malignancies, the FDA approved the clinical use of various DNMTi and HDACi. However, both DNMTi and HDACi as monotherapy have been ineffective in most solid tumors. Nevertheless, because of their strong immunomodulatory effects, promising results are expected from their combination with ICI [32]. In this perspective, a new therapeutic frontier could be represented by a more rational use of EPIi, alone or in combination, to potentiate IFN-I therapeutic activities. The combination of EPIi with IFN-I is supported by some preclinical data [33]. The potential complementary antitumor activity of EPIi and endogenous or exogenous IFN-I need to be further investigated, taking into account key unanswered variables, such as the right sequence of treatments or unwanted immune-suppressive effects that may occur.

## 3. IFN-I in Antitumor Therapies Targeting Cancer Stem Cells (CSC)

**Figure 2:**
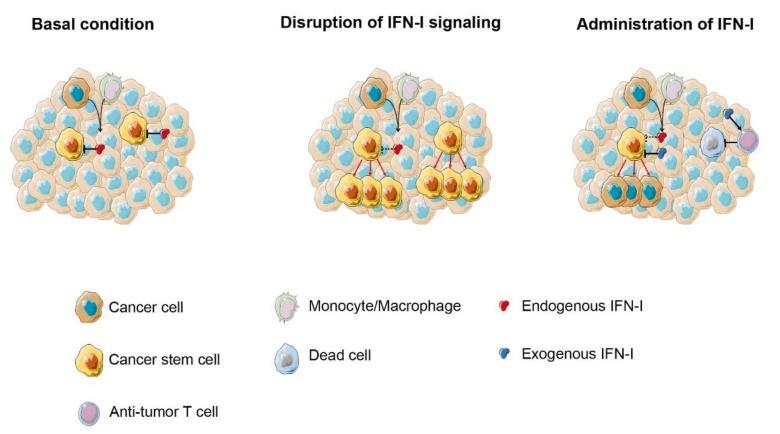
IFN-I exerts an inhibitory effect on growth and persistence of CSC. (**Left**) basal levels of endogenous IFN-I produced by either cancer cells and/or tumor infiltrating immune cells control Cancer Stem Cells (CSC) proliferation and differentiation (as demonstrated in CML and breast cancer). (**Center**) disruption of IFN-I signaling leads to the increased proliferation and migration of CSC. (**Right**) low-dose exogenous IFN-I administration can have a double effect on CSC by favoring differentiation over self-renewal and activating immune response against CSC.

Recent studies highlight an unexpected relationship between IFN-I and CSC that opens up perspectives for the design of novel antitumor therapies (Figure 2).

According to the CSC theory, as normal stem cells renew organs and tissues, a minority of cancerous cells within an entire tumor hold the ability to reproduce themselves and sustain cancer growth, thus acting as a reservoir of cancer cells relapsing after surgery, chemotherapy, or radiotherapy. In CML, data supporting the effect of IFN-α in limiting CML stem cells derive primarily from clinical experience. Notably, while Imatinib and other Tyrosine Kinase Inhibitors (TKI) proved to be superior in inducing complete hematological remission and cytogenetic response with minimal toxicity, IFN-α treatment led to continuous cytogenetic remission even after the cessation of treatment, thus suggesting a curative effect [34,35,36,37], possibly due to both immunomodulation and restriction of stem cells [38]. Therefore, we believe that combination therapy of a low dose IFN-I with TKI in CML therapy deserves further clinical evaluation.

The involvement of IFN-I in the restraint of CSC was also recently reported by our group and others in breast cancer models [7,39,40]. In Her2/Neu transgenic mice, an impaired IFN-I signaling resulted in an increased amount of breast CSC during spontaneous carcinogenesis. Besides being consistent with experimental data showing a close relationship between IFN-I and CSC [39,41], these results corroborate clinical data showing that an impaired IFN-I signaling correlates with a worse clinical outcome and a lower therapy responsiveness in cancer [6,12,39]. Accumulating evidence indicates a specific role for IFN-β, even when administered at a low dose, in counteracting CSC stemness in breast cancer, not only through immunomodulating mechanisms, but also by transcriptionally controlling CSC differentiation [40,42].

Further steps in IFN-I research need to conclusively dissect the role of endogenous and exogenous IFN-I on the biology of CSC in different clinical settings. As for breast cancer, IFN-β seems the best candidate to be tested in clinical protocols aimed at preventing tumor recurrence in adjuvant settings. We envisage a continuous (or semi-continuous) treatment with low doses of the cytokine, which should guarantee the restoration of basal levels of the cytokine that the tumor might suppress.

## 4. IFN-I for Cancer Vaccination

### 4.1. IFN-I as Immune Adjuvant for Cancer Vaccines

The main research challenges the development of cancer vaccines, include the identification of optimal strategies for reverting the immune suppression in cancer patients and increasing the immune response to tumor antigens. Studies in mouse and human models performed by our group and others over the last two decades have suggested that IFN-I can act as a powerful vaccine adjuvant by multiple mechanisms, including *in vivo* differentiation/activation of DC [43] and references therein). Notably, IFN-I was used in pilot studies as a vaccine adjuvant in infective [44] and neoplastic human diseases (references reviewed in [43]). We showed that in advanced melanoma patients the vaccination with melanoma peptides, combined with low dose IFN-α given locally and concomitantly, resulted in enhanced specific CD8 + T cells and monocyte/DC precursor activation [45], resulting in an encouraging clinical benefit in the absence of substantial toxicity (Urbani et al., submitted). In both these two studies in patients with advanced melanoma, IFN-α 2b (3–6 million units) was administered s.c. at the time of repeated i.d. injections of the melanoma peptides, with the main rationale of inducing DC activation, thus promoting an antitumor immune response. We believe that the development of a more effective cancer vaccine should consider the potential contribution of IFN-I, as well as of IFN-I-inducers used as a local immune adjuvant.

### 4.2. IFN-α in DC-Based Combination Immunotherapy

**Figure 3:**
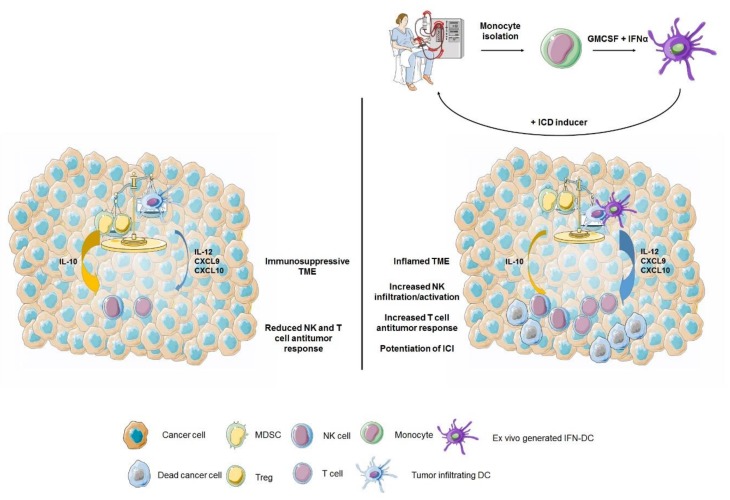
IFN-DC for in situ vaccination. The balance between immune activating vs. immunosuppressive cells/signals affects the antitumor function of immune effector cells. In immunosuppressed tumors (**left**), myeloid derived suppressor cells (MDSC), and regulatory T cells (Treg) overcome immune activating signals released by tumor infiltrating DC, by both direct inhibitory signals and secreted cytokines (e.g., IL-10), eventually resulting in reduced antitumor activity of both the effector T cells and NK cells. (**Right**) in situ vaccination with ex vivo generated IFN-DC (**top**), combined with immunogenic cell death (ICD) inducers to ensure the release of tumor antigens, stimulates DC cross-presentation and tumor-specific T cells generation. Additionally, IFN-DC can overrun immunosuppressive cells and signals by secreting a high amount of immune activating cytokine IL-12 and T cell attracting chemokines (CXCL9 and CXCL10), thus subverting the tumor microenvironment into a more inflamed and immune active one.

An ensemble of data published over the last two decades have shown that IFN-I are important factors for inducing a rapid differentiation and activation of DC in both mouse and human models (reviewed in [43]) and that IFN-DC interactions can play key roles in the antitumor immune response [46,47]. Of note, monocytes short-term cultured with GM-CSF and IFN-α generate DC, named IFN-DC, with a unique attitude to take-up tumor apoptotic bodies and induce a potent tumor specific T cell immunity [48,49,50,51]. We exploited the use of these cells in two pilot clinical trials (in melanoma and follicular lymphoma) in combination with death-inducing agents aiming at in situ vaccination and the overcoming of immunosuppressive signals [52,53]. Interestingly, we observed activation of the anti-tumor response and objective clinical response in a large portion of patients, thus pointing to this approach as a valuable tool to increase antitumor response. Notably, recent studies have shown that an effective antitumor response to anti-PD1 antibodies strictly requires the occurrence of intratumoral DC producing IL-12 [54], and well-defined interactions between NK cells and DC in the tumor microenvironment [55]. Of interest, IFN-DC are high producers of IL-12 [56] and, in view of the recent finding on the role of intratumoral IL-12 producing DC in mediating the response to ICI [54], they can represent good candidates for potentiating anti-PD1-based therapies [57]. We envisage therapeutic scenarios where cancer patients are treated with IFN-DC either as unloaded antigen-presenting cells injected intratumorally [57] or as in vitro antigen loaded DC, and subsequently injected with anti-PD1 antibodies or other ICI to increase the antitumor response in selected combination therapies (Figure 3).

## 5. Conclusions

After more than 50 years since the initial demonstration of the antitumor effects of IFN-I in mice, we are still discovering new and important functions of these cytokines in cancer, suggesting novel rationales and modalities for their clinical use. Notably, the use of old drugs, either for new therapeutic uses or with qualitatively new modalities, exhibits advantages over new drugs approval, in terms of costs and impact on public health systems, thanks to reduced costs and time needed for clinical development [58].

**Figure 4:**
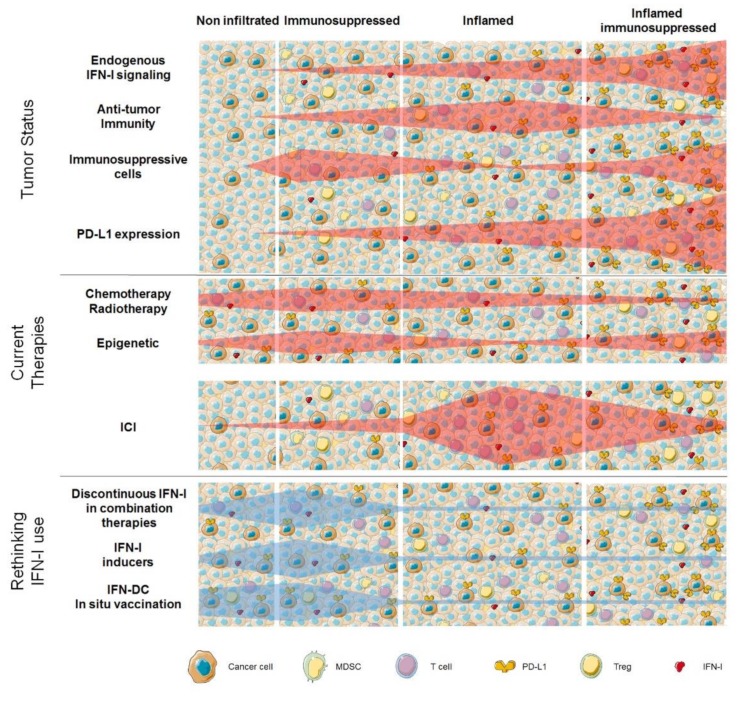
The conclusion. The tumor microenvironment (TME) is characterized by an extremely high inter and intra-tumor heterogeneity (**top**), with tumors showing different levels and forms of immune cell infiltration. The extent of endogenous IFN-I signaling activation plays a key role in regulating quantity and quality of immune cell infiltrate and, as a consequence, tumor PD-L1 expression levels. Absence of IFN-I signaling is typical of non-infiltrated tumors (**left**). Insufficient levels of IFN-I signaling are usually associated with immunosuppressed TME with a high infiltration of MDSC and Treg and low percentages of antitumor T cells. Tumors with intact IFN-I signaling are commonly inflamed with antitumor T cells that can be inhibited by the expression of immune checkpoints (**center**), whereas excessive IFN-I signaling activation is often observed in tumors with exhausted antitumor T cells and activation of immunosuppressive mechanisms (**right**). In light of such heterogeneity, efficacy of current therapies (**middle**) varies depending on tumor status and TME features. In settings with absent or reduced IFN-I signaling, chemotherapy, radiotherapy, and epigenetic drugs can modify TME by reactivating IFN-I signaling, thus resulting in the induction of antitumor response and/or decreased immunosuppression. In case IFN-I signaling is active in TME, ICI can exert its maximum potentials. (**Bottom**) potential windows of action for renewed use of IFN-I (either directly or indirectly) are hypothesized. Exogenous IFN-I or endogenous IFN-I inducers can be timely combined with anti-tumor cytotoxic therapies to induce antitumor immunity or overcome tumor-induced immune suppression. In the case of non-infiltrated tumors, chemotherapy or radiotherapy can induce ICD, thus releasing tumor antigens, which in the context of endogenous-induced or exogenous administered IFN-I can potentiate anti-tumor immune responses. In tumor-induced immunosuppressed TME, activation of IFN-I signaling can reverse immunosuppression. In both cases, IFN-DC-based in situ vaccination can be envisaged to stimulate antitumor immunity or overcome immunosuppression.

Today, combining different immunotherapeutic agents with conventional and novel drugs and treatments, taking into consideration the advances in our understanding of the mechanisms of action, is unanimously considered of great importance for developing more effective and personalized cancer therapies [59]. As shown in Figure 4, levels of expression of endogenous IFN-I concur to the complex balance between TME immune-infiltration vs. immune-suppression, thus depicting different scenarios that differently respond to currently available anticancer therapies.

In our vision, in settings characterized by low or absent endogenous IFN-I signaling a rationale-based rethinking of IFN-I clinical use, mainly based on the transient administration of the cytokine, or IFN-I inducers, or the supplementation of ex vivo-generated IFN-DC, can potentially affect this balance, paving the way to the increased response to ICI. On the other hand, new efforts are necessary to better characterize the mechanisms underlying endogenous IFN-I overexpression in TME, and to develop effective strategies to counterbalance its potentially detrimental effect.

Novel immunotherapies based on activators of the STING pathways or other endogenous IFN related signals (extensively discussed in other reviews [2,13]) are in an early phase of clinical experimentation and may represent a highly valuable approach for a localized activation of the endogenous IFN-I system to be exploited in cancer therapy. Recent advances have been made also in in vivo targeted delivery of IFN-I by Ab conjugates [60,61]. Although the selective targeting of IFN to either tumor cells or immune cells may cause a more specific effect, it appears to be difficult to determine the ideal cell type as the optimal and unique target of IFN for all the different clinical settings.

We hope that this article has delivered the main message that IFN-I are not yet “dead drugs” in clinical oncology. We predict that recombinant IFN-I, together with new products affecting endogenous IFN production or response, will find an important place in future combination therapies of cancer, provided that further preclinical and clinical studies will address the critical issues regarding optimal modalities for delivering the right amount of the cytokines at the right time, in the right place, and in the right patients.
